# *Community Eye Health Journal* – promoting improvement in eye health for over 20 years

**Published:** 2012

**Authors:** Elmien Wolvaardt Ellison

**Affiliations:** Editor, Community Eye Health Journal, International Centre for Eye Health, London School of Hygiene and Tropical Medicine, Keppel Street, WC1E 7HT, UK. Email: editor@cehjournal.org

**Figure:**
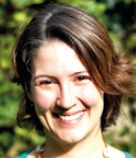
Elmien Wolvaardt Ellison

The *Community Eye Health Journal* was established in 1988 and is published by the International Centre for Eye Health, based at the London School of Hygiene and Tropical Medicine. It has editions in French, Chinese, and Spanish, and there is a special edition for India. Paper copies of the journal, in all four languages, are sent to over 35,000 people in 183 countries (see map).

We would like to thank everyone who completed our recent reader survey. Here is a brief summary of the results.

## Where our readers are

- A total of 1,418 responses were received (5.3% response rate). 59% were from Africa and 32% from Southeast Asia; the remaining 9% were spread across the other regions.
- Half of respondents worked in small towns, villages or rural areas; the other half worked in larger towns or capital cities.
- Two thirds of respondents worked for government, a quarter in the private sector, and the remainder worked for non-governmental organisations.
- Nearly 40% of respondents worked at primary level; 34% at secondary level, and the remainder at tertiary level.

## What our readers do

- The biggest professional group represented were ophthalmic nurses (33%), followed by ophthalmologists (26%) and optometrists (12%; double the number in 2005). Non-eye care specialists made up 29% of the respondents, including nurses, doctors, administrators, pharmacists, researchers, and technicians.
- More than half of respondents had a wider range of responsibilities than those described by their profession. Around 60% reported that community development/outreach, health promotion, and patient counseling were part of their work responsibilities; 40% reported being responsible for programme planning and management, 22% for hospital administration and management, and 14% were also policy makers.

## Access

- A total of 57% of respondents had internet access whenever needed, but around half cited slow speeds, high costs, and lack of know-how as reasons for preferring not to read the journal online. In another part of the survey, respondents described using the paper copy as a teaching aid when educating patients or training students.
- Nearly two thirds of respondents had access to a computer, and 79% had found the Community Eye Health Update CD ‘useful’ or ‘very useful’.

## Impact

- 91% of respondents said they used the journal to teach or educate others, including patients and the community.
- 90% of respondents agreed that the journal had improved and/or supported their work.
- 80% said that something they read in the journal had led them to change their clinical practice or management of patients.
  **Figure:**
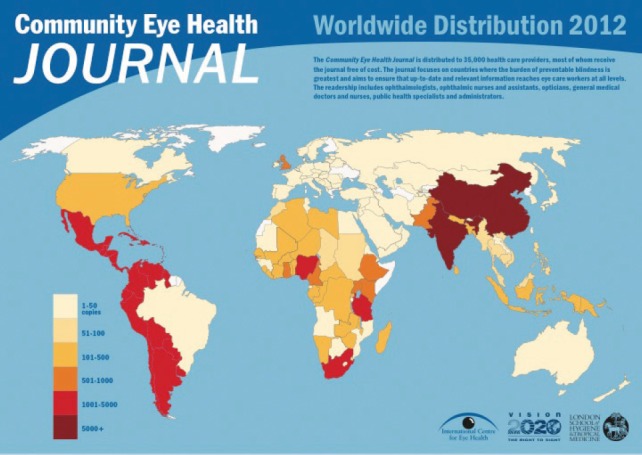
Over 35,000 people in 183 countries receive copies
- The vast majority of respondents (89%) worked directly with patients; they had contact with an average of 60–79 patients per week.
- 80% agreed that the journal had motivated them to reach out to the community, 75% that it had changed the way they conducted health education, and 70% agreed that it had changed the way they talked to patients, stimulated them to talk to non-eye care colleagues, and motivated them to stay in eye care.
- Respondents passed on the journal to an average often other readers each.

We are encouraged by the positive response to the journal and appreciate the many helpful suggestions for future themes we received.

With thanks to Prof Allen Foster, Prof Clare Gilbertl, Anita Shah, Sally Parsley, and George TH Ellison DSc.

