# Supplementary material for: Community Eye Health Journal – promoting improvement in eye health for over 20 years
Source: Community Eye Health. 2012;25(78):35. (PMC3483611)
Supplement: Related texts

# Brief report: Putting patients at the centre of eye care

**Published:** 2012

**Understanding what patients think (page 22)**

- Visit www.institute.nhs.uk and type ‘patient perspectives’ in the search box
- Read more about KAP surveys in on **www.uniteforsight.org/global-health-university/survey-methodologies**
- Family planning clinics in Latin America successfully used exit interviews to improve quality of care and patient satisfaction. **www.guttmacher.org/pubs/journals/2606300.html**
- Using KAPs to plan DR services: a report from LAICO. **http://laico.org/v2020resource/files/KAPStudyMethodology.pdf** (PDF, 410kB)

**Improving patient flow (page 31)**

- Community Eye Health J, Vol. 23 No. 73. **Equipment for eye care**
- Community Eye Health J, Vol. 23 No. 74. **Ten years to VISION 2020: why information matters**
- Community Eye Health J, Vol. 24 No. 76. **Instruments and consumables**
- Visit **www.institute.nhs.uk** and search for each of the following (by typing the term into the search box), in turn: ‘patient flow’, ‘bottlenecks’, ‘process mapping’, and ‘value stream mapping’.
- Read case studies on patient flow from the UK, including ones in eye care. **www.carebydesign.org/files/no_delays_achiever_case_studies.pdf** (PDF, 1.1MB)
- How Aravind Eye Care Systems in India improves patient flow. **www.accessh.org/publication/Article/14**
